# A Great Opportunity for Good

**Published:** 1887-02-19

**Authors:** 


### A GREAT OPPORTUNITY FOR GOOD.

WE received on the 14th inst., a five-pound Bank of England note, No. $\frac{98}{v}$ 82,715, as "a small contribution towards ambulances for conveying patients from the home to the hospital," from "an individual who feels jolting." This is a very acceptable valentine, and we shall hold the money in trust, in the hope that sufficient funds may one day be raised to establish a complete system of horse ambulance throughout London. The cost of establishing such a system would be a mere trifle to many wealthy people, and we know of no such useful means of doing great practical good to the people at a relatively small cost. Will no one of their abundance establish such an organisation in this year of jubilee

